# Atrial Fibrillation Detection With an Analog Smartwatch: Prospective Clinical Study and Algorithm Validation

**DOI:** 10.2196/37280

**Published:** 2022-11-04

**Authors:** David Campo, Valery Elie, Tristan de Gallard, Pierre Bartet, Tristan Morichau-Beauchant, Nicolas Genain, Antoine Fayol, David Fouassier, Adrien Pasteur-Rousseau, Etienne Puymirat, Julien Nahum

**Affiliations:** 1 Withings Issy Les Moulineaux France; 2 Intensive Care Unit Centre Cardiologique du Nord Sainte-Denis France; 3 Cardiology Intensive Care Unit Hopital Europeen Georges Pompidou Paris France; 4 Institut Coeur Paris Centre Turin Paris France; 5 Institut Coeur Paris Centre Floréal Bagnolet France

**Keywords:** atrial fibrillation, mobile health, mHealth, diagnosis, electrocardiogram, ECG, smartwatch, smart technology, wearable, cardiology, cardiac, heart failure, heart disease, cardiovascular, morbidity, automatic detection, algorithm, physician, sensor, digital health

## Abstract

**Background:**

Atrial fibrillation affects approximately 4% of the world’s population and is one of the major causes of stroke, heart failure, sudden death, and cardiovascular morbidity. It can be difficult to diagnose when asymptomatic or in the paroxysmal stage, and its natural history is not well understood. New wearables and connected devices offer an opportunity to improve on this situation.

**Objective:**

We aimed to validate an algorithm for the automatic detection of atrial fibrillation from a single-lead electrocardiogram taken with a smartwatch.

**Methods:**

Eligible patients were recruited from 4 sites in Paris, France. Electrocardiograms (12-lead reference and single lead) were captured simultaneously. The electrocardiograms were reviewed by independent, blinded board-certified cardiologists. The sensitivity and specificity of the algorithm to detect atrial fibrillation and normal sinus rhythm were calculated. The quality of single-lead electrocardiograms (visibility and polarity of waves, interval durations, heart rate) was assessed in comparison with the gold standard (12-lead electrocardiogram).

**Results:**

A total of 262 patients (atrial fibrillation: n=100, age: mean 74.3 years, SD 12.3; normal sinus rhythm: n=113, age: 61.8 years, SD 14.3; other arrhythmia: n=45, 66.9 years, SD 15.2; unreadable electrocardiograms: n=4) were included in the final analysis; 6.9% (18/262) were classified as Noise by the algorithm. Excluding other arrhythmias and Noise, the sensitivity for atrial fibrillation detection was 0.963 (95% CI lower bound 0.894), and the specificity was 1.000 (95% CI lower bound 0.967). Visibility and polarity accuracies were similar (1-lead electrocardiogram: P waves: 96.9%, QRS complexes: 99.2%, T waves: 91.2%; 12-lead electrocardiogram: P waves: 100%, QRS complexes: 98.8%, T waves: 99.5%). P-wave visibility accuracy was 99% (99/100) for patients with atrial fibrillation and 95.7% (155/162) for patients with normal sinus rhythm, other arrhythmias, and unreadable electrocardiograms. The absolute values of the mean differences in PR duration and QRS width were <3 ms, and more than 97% were <40 ms. The mean difference between the heart rates from the 1-lead electrocardiogram calculated by the algorithm and those calculated by cardiologists was 0.55 bpm.

**Conclusions:**

The algorithm demonstrated great diagnostic performance for atrial fibrillation detection. The smartwatch’s single-lead electrocardiogram also demonstrated good quality for physician use in daily routine care.

**Trial Registration:**

ClinicalTrials.gov NCT04351386; http://clinicaltrials.gov/ct2/show/NCT04351386

## Introduction

Atrial fibrillation is the most common form of arrhythmia in the world—it affects 8 million people in Europe and 5 million people in the United States [1]. Experts believe that the number of patients with atrial fibrillation will increase in the next few years to one-quarter of middle-aged adults in the United States and in Europe [2,3]. Despite improvements in its management, atrial fibrillation remains one of the major causes of stroke, heart failure, sudden death, and cardiovascular morbidity in the world [3,4].

Atrial fibrillation is associated with a 5-fold increase in the risk of stroke [5-9]. Large randomized controlled trials [10,11] are underway to evaluate stroke prevention by using anticoagulation treatment in patients with subclinical atrial fibrillation. Asymptomatic patients represent 32.6% to 39.4% of patients in large international registries [12,13]. Thus subclinical atrial fibrillation could represent approximately one-third of the atrial fibrillation population [14] and is admittedly found frequently among older adults [15,16].

Traditionally, atrial fibrillation is diagnosed using an electrocardiogram (ECG), a Holter monitor worn for 24 to 48 hours, an event recorder monitoring heart activity for several weeks, or implanted pacemakers or defibrillators with an atrial lead. There is trade-off in efficacy of detection of paroxysmal atrial fibrillation between short- and long-term monitoring.

Given the increasing number of patients with asymptomatic atrial fibrillation, new simple and efficient diagnostic devices are important supplements to traditional methods to allow early diagnosis [17], screening [18], or management [19]. It is therefore crucial that these devices be evaluated in clinical studies in comparison with standard 12-lead electrocardiography [20].

We aimed to validate the diagnostic performance (ie, classification into atrial fibrillation or normal sinus rhythm) and safety of a single-channel ECG smartwatch (ScanWatch, Withings) and its associated software (Scan Monitor, Withings Inc), in the detection of atrial fibrillation in comparison with reference diagnoses made by independent blind cardiologists using simultaneously recorded 12-lead ECG.

## Methods

### Study Design and Population

We conducted a prospective nonrandomized open-label comparative multicenter study. Cardiology in-patients and out-patients were consecutively screened from 4 sites in Paris, France. Inclusion criteria were male or female patients aged 18 years or older with atrial fibrillation or sinus rhythm, recruited with a 1:1 ratio. Patients with pacemakers, who were physically incapable of wearing a watch on their wrist, with linguistic or mental incapacity that precluded signing a written informed consent form, or vulnerable individuals (as defined by French regulation) were not included. Eligible patients were informed and provided consent prior to any study-related procedure.

### Study Procedures

#### Data Collection

For each patient, simultaneous 30-second single-lead ECGs were recorded with ScanWatch with embedded software (Scan Monitor) and 12-lead ECGs recorded with Schiller Cardiovit FT1 electrocardiograph (CE marked and FDA cleared [21]).

ScanWatch uses 3 dry electrodes to record a 30-second single-lead ECG that is similar to lead I of a traditional 12-lead ECG. A real-time signal captured with the watch is streamed to a smartphone app (Health Mate, Withings; for Android and iOS), stored, exported in PDF format, and classified into 4 categories—atrial fibrillation, normal sinus rhythm, noise, or other—by the proprietary algorithm. The classification is performed on 30-second recordings using features related to the visibility of P waves and the irregularity of R-R intervals. Algorithm classifications were kept on Withings servers, and investigators were blinded from it. Data from the 12-lead ECG were exported using DICOM/HL7 ECG Waveform Export software (SemaServer, version 19.02; Schiller) in accordance with manufacturer’s instructions. Patient information and data were collected and reported by site staff on the study case report form.

#### Data Review

The 12-lead reference ECGs and single-lead smartwatch ECGs were independently reviewed by blinded, board-certified cardiologists. Each recording was reviewed by 3 reviewers. The reviewers were instructed to classify each recording into one of the following categories: (1) normal sinus rhythm, (2) atrial fibrillation, (3) supraventricular tachycardia, (4) abnormal rhythm, such as frequent premature atrial contractions, frequent premature ventricular contractions, atrial flutter, ventricular tachycardia, ventricular fibrillation, second-degree atrioventricular block type I, second-degree atrioventricular block type II, third-degree atrioventricular block, and other, or (5) uninterpretable (ie, a classification cannot be made as the strip is not adequate for reading). If there were multiple rhythms, reviewers reported all the rhythms, then classified the recording in one of the above classes with justification. If there was a discrepancy between reviewers’ classifications, the diagnosis of the majority was retained. For the 3 primary rhythms (normal sinus rhythm, atrial fibrillation, and supraventricular tachycardia), if classifications differed, the final classification was determined by consensus.

Reviewers’ classifications were compared to the algorithm’s automatic classification of the single-channel strips collected with the smartwatch to assess atrial fibrillation detection performance. The software classified the smartwatch strips as normal sinus rhythm, atrial fibrillation, Noise, or other arrhythmia (supraventricular tachycardia and other abnormal rhythm reference diagnoses were pooled).

To assess the quality of the single-lead ECG signals generated by the smartwatch and whether the smartwatch could be used by cardiologists in clinical practice, cardiologists assessed the visibility and polarity of P, QRS, and T waves; measured the durations of PR, QRS, and QT intervals; and measured heart rate. An additional reviewer selected a well-defined beat to be later used by the reviewers for secondary outcome measures.

Diagnoses made by the reviewers from the single-channel strips generated by the smartwatch were compared with those from the 12-lead ECG.

### Statistical Analysis

The primary outcomes were sensitivity and specificity of atrial fibrillation detection. Sensitivity and specificity were calculated, first, by considering all available categories, and second, by excluding Other and Noise. The reason for the second calculation is that the device is primarily intended to discriminate between atrial fibrillation and normal sinus rhythm. Calculated sensitivities and specificities are reported with their lower confidence interval bounds; their positive and negative likelihood ratios were also calculated.

Continuous variables were expressed as mean and standard deviation, or median and range, while categorical variables were expressed as numbers and proportions. Exact 95% confidence intervals of proportions were calculated with the Clopper-Pearson method. Results for the 4 classes (atrial fibrillation, normal sinus rhythm, Noise, and Other) are presented with a 4×4 confusion matrix.

Baseline characteristics were compared using the *t* test for sample means for normal distributions, Mann-Whitney test for sample medians otherwise, and Fisher exact test for proportions. A Shapiro-Wilk test was used to test normality.

Cardiologist agreement was measured using Cohen κ and average accuracy in comparison with the consensus diagnosis.

Sample size was calculated for sensitivities and specificities >0.9 and a statistical power >90%. All statistical tests were 2-sided with a statistical significance threshold at .05. Analyses were performed with Python (version 3.6.8) scikit-learn (version 0.23.2), statsmodels (version 0.12.2), and scipy (version 1.5.2) toolkits.

### Ethics

This study was conducted in compliance with Good Clinical Practice and 1964 Declaration of Helsinki and subsequent amendments [22]. The study was approved by the French *Comité de Protection des Personnes* *CPP Sud-Est IV* (19.06.28.65727) and registered (ClinicalTrials.gov, NCT04351386).

## Results

### Population Characteristics

**Figure 1:**
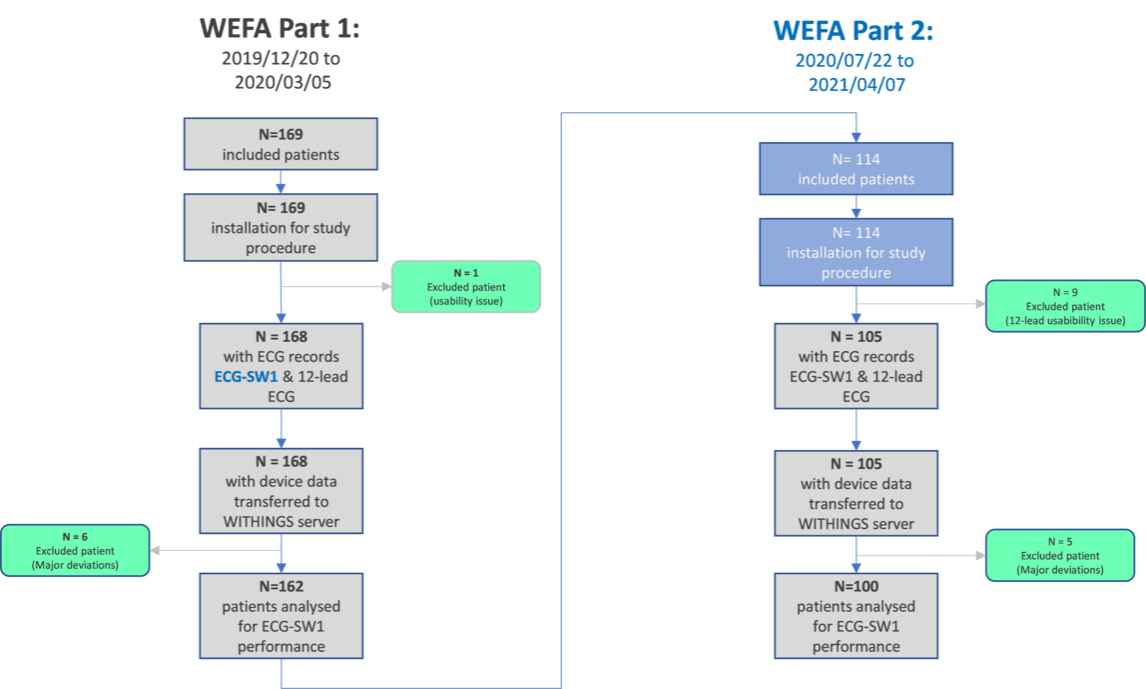
Study flowchart. ECG: electrocardiogram.

Between December 2019 to April 2021, 283 patients were enrolled in the study; however, 10 patients prematurely discontinued their participation, and 11 patients were excluded, which resulted in an analysis set of 262 patients (Figure 1). Patients characteristics (men: n=160, women: n=102; age: mean 67.7 years, SD 14.8, BMI: mean 27.5, SD 5.7 kg/m^2^) are presented in Table 1.

**Table 1:** Baseline patient characteristics.

Characteristic			All patients (n=262)		Atrial fibrillation (n=100)		Normal sinus rhythm (n=113)		Other arrhythmias (n=45)		Unreadable ECG^a^ (n=4)
Age (years), mean (SD)			67.7 (14.8)		74.3 (12.3)		61.8 (14.3)		66.9 (15.2)		78.8 (12.5)
Height (cm), mean (SD)			169.2 (9.2)		168.8 (9.4)		169.7 (9.2)		169.3 (9.0)		163.8 (7.8)
Weight (kg), mean (SD)			78.9 (17.6)		79.1 (19.1)		79.1 (15.1)		78.9 (20.1)		67.8 (18.7)
BMI^b^ (kg/m^2^), mean (SD)			27.5 (5.7)		27.6 (6.0)		27.5 (5.1)		27.5 (6.4)		25.3 (6.5)
**Sex, n (%)**											
	Female	102 (38.9)		42 (42.0)		39 (34.5)		18 (40.0)		3 (75.0)	
	Male	160 (61.1)		58 (58.0)		74 (65.5)		27 (60.0)		1 (25.0)	
**Patient follow-up at site during study procedure, n (%)**											
	In-patient	205 (78.2)		91 (91.0)		72 (63.7)		38 (84.4)		4 (100)	
	Out-patient	57 (21.8)		9 (9.0)		41 (36.3)		7 (15.6)		0 (0.0)	
**Cardiovascular medical history, n (%)**											
	Atrial fibrillation	122 (46.6)		91 (91.0)		11 (9.7)		19 (42.2)		1 (25.0)	
	Valvular heart disease with or without intervention before inclusion	46 (17.6)		25 (25.0)		12 (10.6)		8 (17.8)		1 (25.0)	
	Coronary artery disease with or without coronary artery bypass grafting before inclusion	72 (27.5)		27 (27.0)		30 (26.5)		14 (31.1)		1 (25.0)	
	Heart failure	26 (9.9)		20 (20.0)		3 (2.7)		3 (6.7)		0 (0.0)	
	Myocardial infarction or ischemic cardiopathy	31 (11.8)		17 (17.0)		9 (8.0)		5 (11.1)		0 (0.0)	
	Transient ischemic attack or stroke	12 (4.6)		9 (9.0)		2 (1.8)		1 (2.2)		0 (0.0)	
	Peripheral arterial obstructive disease	18 (6.9)		7 (7.0)		8 (7.1)		3 (6.7)		0 (0.0)	
	Abdominal aortic aneurysm	4 (1.5)		1 (1.0)		3 (2.7)		0 (0.0)		0 (0.0)	
**Cardiovascular risk factors, n (%)**											
	Hypertension	135 (51.5)		60 (60.0)		51 (45.1)		23 (51.1)		1 (25.0)	
	Dyslipidemia	90 (34.4)		36 (36.0)		39 (34.5)		15 (33.3)		0 (0.0)	
	Former or current smoker	77 (29.4)		28 (28.0)		36 (31.9)		13 (28.9)		0 (0.0)	
	Overweight	70 (26.7)		27 (27.0)		32 (28.3)		9 (20.0)		2 (50.0)	
	Obesity	42 (16.0)		14 (14.0)		22 (19.5)		6 (13.3)		0 (0.0)	
	Diabetes	57 (21.8)		23 (23.0)		23 (20.4)		11 (24.4)		0 (0.0)	
**Position during ECG recording, n (%)**											
	Supine	115 (43.9)		44 (44.0)		50 (44.2)		18 (40.0)		3 (75.0)	
	Sitting	147 (56.1)		56 (56.0)		63 (55.8)		27 (60.0)		1 (25.0)	
	Wrist										
	Left	179 (68.3)		66 (66.0)		82 (72.6)		30 (66.7)		1 (25.0)	
	Right	83 (31.7)		34 (34.0)		31 (27.4)		15 (33.3)		3 (75.0)	
**Skin type, n (%)**											
	White	204 (77.9)		82 (82.0)		84 (74.3)		35 (77.8)		3 (75.0)	
	Mediterranean/Arabic	35 (13.4)		15 (15.0)		13 (11.5)		6 (13.3)		1 (25.0)	
	Black	23 (8.8)		3 (3.0)		16 (14.2)		4 (8.9)		0 (0.0)	

^a^ECG: electrocardiogram.

^b^BMI: body mass index.

The atrial fibrillation group (mean 74.3 years, SD 12.3) was significantly older than the normal sinus rhythm group (mean 61.8 years, SD 14.3, *P*<.001) and other arrhythmia groups (mean 66.9 years, SD 15.2, *P*=.002). Similarly, the proportion of in-patients was higher in the atrial fibrillation group (91/100, 91%) than those in the normal sinus rhythm (72/113, 64%) and other arrhythmia groups (38/45, 84%). Out-patients (age: mean 59.1 years) were significantly younger (*P*<.001) than in-patients (age: mean 70.1 years).

Among all cardiovascular risk factors, hypertension was the most represented in all subgroups (atrial fibrillation: 60/100, 60%; normal sinus rhythm: 51/113, 45%; other arrhythmia: 23/45, 51%).

Cardiovascular arterial disease was the most represented cardiovascular risk with similar prevalences across the 3 subgroups (atrial fibrillation: 27/100, 27%; normal sinus rhythm: 30/113, 27%; other arrhythmia: 14/45, 31%). Only 9 patients (9/100, 9%) in the atrial fibrillation subgroup did not have a history of atrial fibrillation before participating in the study. No adverse events occurred during the study.

### Automatic Atrial Fibrillation Detection Performance

Supraventricular tachycardia was not diagnosed by any of the independent cardiologist reviewers using 12-lead ECGs. No consensus meetings were needed. The average accuracy of the 3 cardiologists was 0.92 (mean Cohen κ=0.88).

Four reference ECG were labeled as Noise, 2 of which were classified as Noise by ECG Monitor, and 2 as atrial fibrillation. ECG Monitor classified 6.9% (18/262) of the recordings performed with the watch as Noise, but none was from a patient with normal sinus rhythm (Table 2). When considering all 4 categories, the sensitivity to detect atrial fibrillation was 0.77 (95% CI lower bound 0.675), and the specificity was 0.965 (95% CI lower bound 0.912). Of the 113 normal sinus rhythm diagnoses based on the 12-lead reference ECG, only 1 (0.85%) was classified as Noise by the algorithm, and 13 (13.0%) patients diagnosed with atrial fibrillation based on the 12-lead reference ECG were classified as Noise by the algorithm. When excluding the categories *other arrhythmia* and *Noise* from the calculation, the sensitivity was 0.963 (95% CI lower bound 0.894), and the specificity was 1.000 (95% CI lower bound 0.967). Inconclusive measurements (Other and Noise) occurred more frequently in patients >65 years old (odds ratio [OR] 4.34, 95% CI 1.25-15.09; *P*=.02) and in patients with previously diagnosed atrial fibrillation (OR 3.75, 95% CI 1.43-9.87; *P*=.007). While not statistically significant, in-patients and patients with hypertension or valvular heart disease tended to have more inconclusive ECGs.

**Table 2:** Algorithm classification (1-lead ECG^a^) versus cardiologist diagnosis (12-lead ECG).

Algorithm classification (Smartwatch 1-lead ECG)	Cardiologist diagnosis from 12-lead ECG				
	Normal sinus rhythm, n	Atrial fibrillation, n	Other, n	Noise, n	Total, n
Normal sinus rhythm	109	3	9	0	121
Atrial fibrillation	0	77	11	2	90
Other	3	7	23	0	33
Noise	1	13	2	2	18
Total	113	100	48	4	262

^a^ECG: electrocardiogram.

### ECG Signal Quality

#### Diagnostic Accuracy

The sensitivity for detecting atrial fibrillation was 0.89; the specificity was 0.912 (Table 3). The average accuracy between the 3 cardiologists reading single-channel recordings from the smartwatch was 0.785, and the average Cohen κ was 0.675, which reflected strong agreement between reviewers.

**Table 3:** Cardiologist diagnosis (1-lead ECG^a^ vs 12-lead ECG).

Cardiologist diagnosis from Smartwatch 1-lead ECG	Cardiologist diagnosis from 12-lead ECG				
	Normal sinus rhythm, n	Atrial fibrillation, n	Other, n	Noise, n	Total, n
Normal sinus rhythm	103	0	1	0	104
Atrial fibrillation	2	89	17	2	110
Other	3	2	20	0	25
Noise	5	9	7	2	23
Total	113	100	45	4	262

^a^ECG: electrocardiogram.

#### Visibility and Polarity

P-wave visibility accuracy was 99% (99/100) in patients with atrial fibrillation and 95.7% (155/162) when excluding patients with atrial fibrillation (Table 4).

**Table 4:** Cardiologist review of 1-lead electrocardiogram (ECG) versus 12-lead ECG: P-wave, T-wave, and QRS-complex visibility.

			Identified (n) and accuracy (%) (n=262)
**Visibility**			
	P wave	254 (96.9)	
	QRS complex	260 (99.2)	
	T wave	239 (99.5)	
**Polarity^a^**			
	P wave	88 (100)	
	QRS complex	254 (98.8)	
	T wave	183 (99.5)	

^a^n=88, n=257, and n=184 for P wave, QRS complex, and T wave, respectively.

#### Interval Durations

Except for QT intervals, mean difference absolute values for difference in PR duration and QRS width were less than 3 ms and more than 97% of these differences were less than 40 ms (Table 5).

**Table 5:** Interval duration differences between the algorithm’s and cardiologists’ assessments.

	Duration difference (ms), mean (SD)	Difference <40 ms, n/N (%)
PR duration	–2.79 (17.25)	92/94 (97.9)
QRS width	–0.46 (18.91)	245/252 (97.2)
QT duration	7.13 (26.30)	161/182 (88.5)

#### Heart Rate

**Figure 2:**
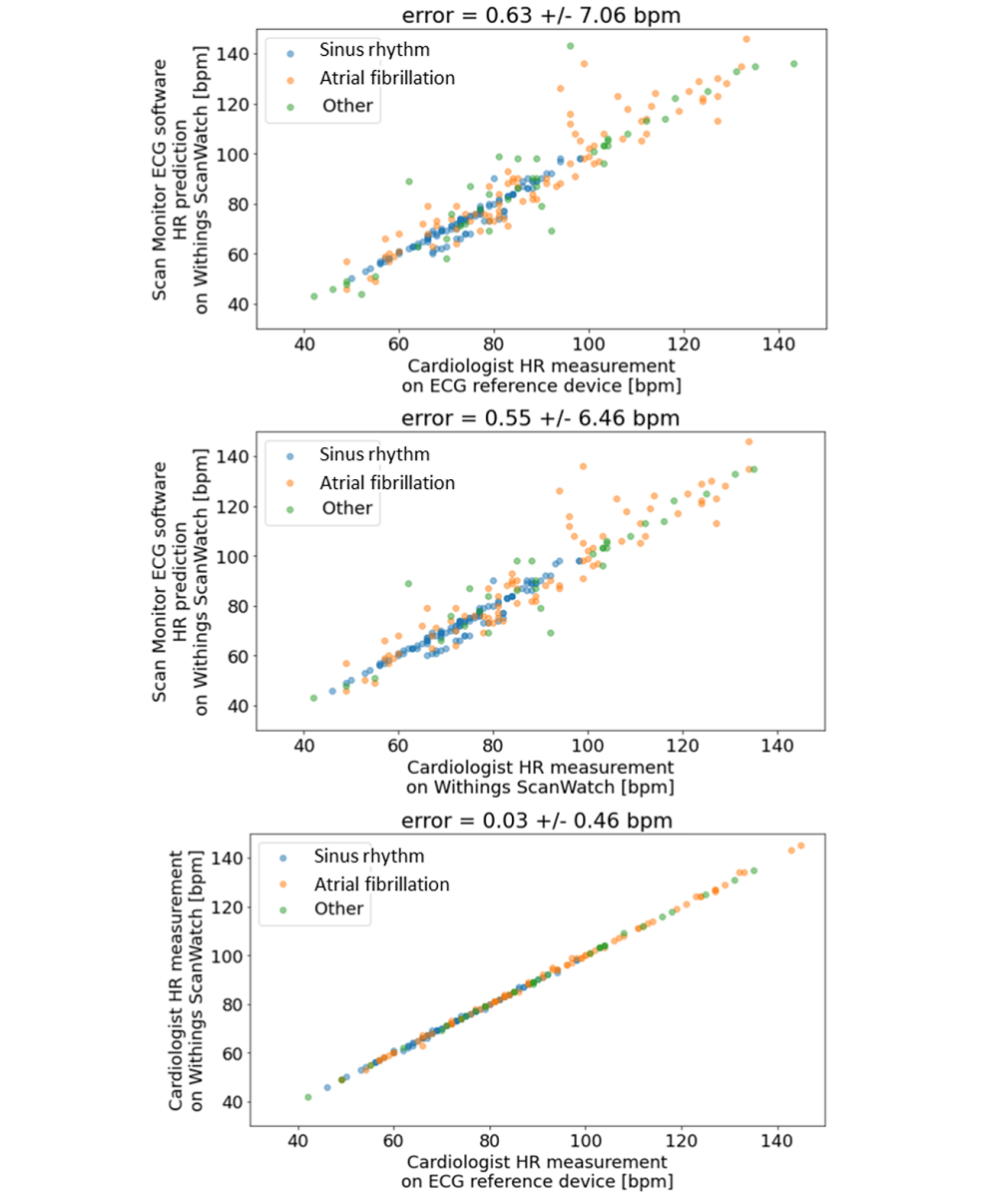
Heart rate calculations by the algorithm versus those by cardiologists. ECG: electrocardiogram; HR: heart rate.

For reviewers’ assessments of heart rate, 1-lead versus 12-lead ECG had the smallest difference (mean 0.03 bpm, SD 0.46). The largest difference was observed between the heart rate calculated by the algorithm compared with that by cardiologists based on 12-lead ECG (mean 0.63 bpm, SD 7.06). The mean difference between heart rates calculated by the algorithm and those by cardiologists based on 1-lead ECG was 0.55 bpm (SD 6.46) (Figure 2).

## Discussion

### Study Strengths

Biases were limited by recruiting consecutive patients, taking the recordings simultaneously, centralizing ECG review with independent blinded reviewers, and standardizing the review process. Participants were in- and out-patients from cardiology services with multiple comorbidities. None was familiar with the device beforehand.

### Principal Results and Comparison With Prior Work

#### Performance for Automatic Atrial Fibrillation Detection by Scan Monitor Software

Only 4 reference ECGs were deemed unreadable; this may occur for multiple reasons especially movements during the measurements.

The algorithm’s ability to discriminate between atrial fibrillation and normal sinus rhythm was calculated both considering all 4 initial categories (sensitivity 0.770; specificity 0.965) and excluding Other and Noise signals (sensitivity 0.963; specificity 1.000).

Misclassifications for patients normal sinus rhythm or atrial fibrillation were very rare. In particular, the rate of false positives was 2.7% (3/113) for normal sinus rhythm, and no patients with atrial fibrillation were classified as patients with normal sinus rhythm by the algorithm. Most false negatives were misclassifications of other arrhythmias, while false positives were mostly misclassifications of patients with normal sinus rhythm or atrial fibrillation as Other. This is unfortunately associated with the algorithm development process being optimized to accurately identify atrial fibrillation and distinguish atrial fibrillation from normal sinus rhythm. Therefore, automatic classification reliability on patients with arrhythmias other than atrial fibrillation may be decreased.

These results are quite similar to those from studies on other wearable devices, such as the Apple Watch (Apple Inc) [23], the Kardia Band (AliveCor) [24], and devices by other manufacturers [25-28] (Table 6). The Apple study recruited, by far, the most patients: almost twice the number included in our study and 4 times that in [24]. All the devices had high rates of noisy or poor-quality signals (6.9% to 16.6%). For data loss, there was only 1 case (1/283, 0.4%) with ScanWatch, none (0/169, 0%) in the Kardia band study [24], and 7.6% (46/602) in Apple Watch study. Moreover, ScanWatch and Kardia band classified 11.3% (24/213) and 17.2% (29/169), respectively, of atrial fibrillation and normal sinus rhythm signals as other arrhythmias (or as unclassified or inconclusive); Apple Watch had only 2.2% (13/602) of such errors, and consequently, had higher sensitivities and specificities than ScanWatch and Kardia band. Overall, Kardia band was less accurate than ScanWatch and Apple Watch, with ScanWatch being more accurate than the Apple Watch for detecting normal sinus rhythm.

Only sparse data were found for other manufacturers. For Samsung’s ECG monitor, 16.8% of ECG recordings were considered either inconclusive or of poor quality [26], similar to Kardia band and Apple Watch; sensitivity was 98.1%, and specificity was 100%. Similarly, Fitbit ECG app performances were 98.7% and 100% for correctly detecting atrial fibrillation and normal sinus rhythm, respectively [28]. Similar results were published for Amazfit [27,29].

**Table 6:** Performances of commercially available devices with electrocardiogram (ECG) sensors.

					Withings ScanWatch			Apple Watch [23]			Kardia Band [24]			MyDiagno-stick [25]			Samsung ECG Monitor [26]			Amazfit Health Band 1S [27]			Amazfit Cardidoc app [29]			Fitbit ECG app [28]
Patients recruited, n					283			602			169			192			544			401			114			472
**Patients analyzed, n**					262			553			169			192			544			401			114			440
	Noise or unreadable, n (%)			182 (6.9)			49 (8.9)			28 (16.6)			—^a^			— (16.8)			15 (3.74)			2 (1.75)			—	
	Other or unclassifiable, n (%)			33 (12.6)			19 (3.4)			29 (17.2)			—			—			—			—			—	
	**All, n/N (%)**																									
		Sensitivity	77/100 (77.0)			236/277 (85.2)			63/91 (69.2)			—			—/— (87.1)			—			—			—		
		Specificity	109/118 (92.4)			238/263 (90.5)			37/78 (47.4)			—			—/— (82.5)			—			—			—		
	**Atrial fibrillation and normal sinus rhythm, n/N (%)**																									
		Sensitivity	77/80 (96.3)			236/240 (98.3)			63/68 (92.6)			—/— (100)			—/— (98.1)			—/— (96.7)			—/— (88.7)			—/— (98.7)		
		Specificity	109/109 (100)			237/238 (99.6)			37/44 (84.1)			—/— (95.9)			—/— (100)			—/— (98.0)			—/— (100)			—/— (100)		

^a^No data were available.

In addition, the ScanWatch algorithm had lower performance in heart failure (sensitivity 0.800, 95% CI lower bound 0.519), myocardial infarction or ischemic cardiopathy (sensitivity 0.769, 95% CI lower bound 0.462), and peripheral arterial obstructive disease (sensitivity 0.714, 95% CI lower bound 0.290) subgroups in detecting atrial fibrillation and normal sinus rhythm. All these patients had various comorbidities and associated medications. Moreover, 11 out of the 31 patients (35.5%) with a history of myocardial infarction or ischemic cardiopathy were also diagnosed with heart failure (ie, had multiple comorbidities).

In a registry-based study [30] conducted in 136 and 211 European cardiology centers, major ECG abnormalities were present in the majority of patients with heart failure. Out of 1460 patients with heart failure, 1222 had major ECG abnormalities with various patterns across the heart failure types [30], and the Euroheart Failure survey showed that ECG abnormalities were present in 98% of patients with heart failure [31]. Older age and being male were associated with an increased risk of ECG abnormalities, as were history of advanced heart valve disease, chronic kidney disease, signs of heart failure decompensation, and use of diuretics and anticoagulants [31].

ECG abnormalities, such as abnormal rhythm, PR duration >250 ms, QRS interval ≥120 ms, and pathological Q waves can be found in most patients with multiple severe underlying diseases and risks factors. Similar patterns were observed in our study (Table 7). Although patients’ medications were not recorded in our study, specific heart failure ECG abnormality risk factors were recorded (sex, age, valvular heart disease, kidney disease). Patients with heart failure have multiple comorbidities that affect their ECGs, and such a population can present ECG abnormalities that are too complex for our algorithm—this represents an extreme worst-case scenario for the device’s algorithm—these patients alone represent 21.4% (56/262) of the study cohort.

**Table 7:** Patient subgroups (heart failure, peripheral arterial obstructive disease, myocardial infarction, or ischemic cardiopathy).

		All other (n=206)	Heart failure (n=26)	Peripheral arterial obstructive disease (n=18)	Myocardial infarction or ischemic cardiopathy (n=31)	
**Sex, n (%)**						
	Male	124 (60.2)	18 (69.2)	10 (55.6)		20 (64.5)
	Female	82 (39.8)	8 (30.8)	8 (44.4)		11 (35.5)
Age (years), mean (SD)		66.2 (15.5)	75.3 (11.6)	74.3 (8.5)	71.9 (9.8)	
Heart rate (bpm), mean (SD)		82.9 (19.9)	86.6 (23.0)	72.2 (10.9)	75.1 (18.8)	
Valvular heart disease, n (%)		37 (18.0)	8 (30.8)	3 (16.7)	5 (16.1)	
Diabetes, n (%)		42 (20.4)	7 (26.9)	5 (27.8)	6 (19.4)	
QRS length (ms), mean (SD)		96.6 (29.4)	114.8 (35.3)	104.8 (26.4)	103.8 (26.5)	
QRS >120 ms, n (%)		32 (15.5)	10 (38.5)	5 (27.8)	7 (22.6)	

#### 1-Lead ECG Quality Assessment

To the best of our knowledge, this study proposes for the first time several criteria for device signal quality assessment without bias (quantitative assessment) that are based on criteria taken from clinical practice.

In total, cardiologists declared 8.8% (23/262) of the signals as uninterpretable, and 2 patients with atrial fibrillation were classified as normal sinus rhythm (2/100). These misclassifications may be explained by artifacts. However in some cases (eg, paroxystic atrial fibrillation is more difficult to diagnose with a 30-second recording), a confirmatory ECG can improve the reliability of the diagnosis.

Accuracies in the assessment of T-wave, P-wave, and QRS-complex visibilities and polarities were high (over 96%, except for T wave: 91%). The accuracy of PR, QRS, QT interval durations was good. In particular, the standard deviation of the differences fell below 20 ms for PR and QRS time intervals, which is less than half the length of the smallest graduation (1 mm) on a standard paper ECG trace (40 ms). The standard deviation of QT interval difference was slightly higher (SD 26.3 ms).

Because Apple used a different method to assess ECG waveform quality, a comparison of ECG quality between the devices was not possible.

Comparison of cardiologist-measured heart rate on the single-lead and lead I of the 12-lead reference ECG, yielded a 0 bias and a standard deviation of the difference of 0.5 bpm.

The standard deviation between automatic heart rate calculation by ScanWatch and heart rate measurements by cardiologists on lead I of a reference ECG was 7 bpm. This difference is a consequence of the different calculation methods between reviewers (mean over 10 seconds) and the algorithm (median heart rate over 30 seconds). Given the mean heart rate (mean 82.35, SD 19.9 bpm), the mean error between the heart rate measured by the device and that measured by the reviewers on the reference ECG (mean 0.63, SD 7.09 bpm) was considered acceptable (the accuracy of the detected heart rate shall be ±10% or ±5/min, whichever is greater [32]).

### Limitations

Despite good diagnostic performance in discriminating between atrial fibrillation and normal sinus rhythm, arrhythmias other than atrial fibrillation were poorly identified: only 51.1% (23/45) of these signals were correctly classified. This poor performance was expected since, by design, the algorithm was trained to specifically identify atrial fibrillation and normal sinus rhythm. Flutter signals were not negligible in our population (13/45) and were misclassified as atrial fibrillation by the algorithm: they are not easily identifiable on lead I, even for a cardiologist. Nevertheless, it is encouraging that 80.0% (36/45) of Other signals were not classified as normal sinus rhythm. Unfortunately, patients’ medications at the time of the measurements were not recorded: the study lacks information about drugs that may affect cardiac rhythm.

The population enrolled in the study may not reflect the real-world use of the device: the study was conducted in cardiology services, but the device is intended for home use, some of the recordings were performed while patients were supine rather than seated, and no patients were familiar with the device before measurements, which may have increased the amount of noisy data collected.

Reduced accuracy of the machine learning classification algorithm in patients with multiple risk factors and comorbidities may be explained by the fact that data sets used to develop the algorithm did not include such specific populations. As we mentioned, these patients commonly present ECG abnormalities that may affect the algorithm’s performance.

Finally, only a single measurement was recorded per patient in order to limit potential bias and test the device in the worst-case scenario. In real-use conditions, a diagnosis from a cardiologist may improve using a second recording, and recording quality may also improve with patient’s practice.

### Conclusions

ScanWatch, with its embedded software (Scan Monitor), was able to provide high-quality ECG traces that are adequate for clinical diagnosis of atrial fibrillation (ie, accurately discriminate between atrial fibrillation and normal sinus rhythm). Additional studies and additional machine learning work will be needed to increase the algorithm’s performance in distinguishing atrial fibrillation from other arrhythmias.

Such a device offers clinicians the ability to remotely monitor patients at risk of atrial fibrillation in their daily practice with a simple, accurate, and noninvasive wearable device.

## Abbreviations

BMI: body mass index
ECG: electrocardiogram
OR: odds ratio
